# Loss of photosynthetic efficiency in the shade. An Achilles heel for the dense modern stands of our most productive C_4_ crops?

**DOI:** 10.1093/jxb/erw456

**Published:** 2017-01-21

**Authors:** Charles P. Pignon, Deepak Jaiswal, Justin M. McGrath, Stephen P. Long

**Affiliations:** 1 University of Illinois, Carl Woese Institute for Genomic Biology and Departments of Crop Sciences and of Plant Biology, 1206 W Gregory Drive, Urbana, IL 61801, USA; 2 Lancaster Environment Centre, Lancaster University, Lancaster LA1 4YQ, UK

**Keywords:** C_4_ photosynthesis, canopy photosynthesis, corn, crop photosynthesis, crop yield, food security, maize, miscanthus, quantum yield, shade acclimation, planting density.

## Abstract

The wild progenitors of major C_4_ crops grew as individuals subjected to little shading. Today they are grown in dense stands where most leaves are shaded. Do they maintain photosynthetic efficiency in these low light conditions produced by modern cultivation? The apparent maximum quantum yield of CO_2_ assimilation $(\Phi_{CO_{2}max,app}),$ a key determinant of light-limited photosynthesis, has not been systematically studied in field stands of C_4_ crops. $\Phi_{CO_{2}max,app}$ was derived from the initial slope of the response of leaf CO_2_ uptake (*A*) to photon flux (*Q*). Leaf fractional light absorptance (α) was measured to determine the absolute maximum quantum yield of CO_2_ assimilation on an absorbed light basis $(\Phi_{CO_{2}max,abs}).$ Light response curves were determined on sun and shade leaves of 49 field plants of *Miscanthus × giganteus* and *Zea mays* following canopy closure. $\Phi_{CO_{2}max,app}$ and $\Phi_{CO_{2}max,abs}$ declined significantly by 15–27% (*P*<0.05) with canopy depth. Experimentally, leaf age was shown unlikely to cause this loss. Modeling canopy CO_2_ assimilation over diurnal courses suggested that the observed decline in $\Phi_{CO_{2}max,app}$ with canopy depth costs 10% of potential carbon gain. Overcoming this limitation could substantially increase the productivity of major C_4_ crops.

## Highlight

Leaves of two highly productive C_4_ crops lose photosynthetic efficiency in low light as they become shaded by new leaves forming above, costing the crop up to 10% of potential productivity.

## Introduction

In modern intensive systems, crops form dense canopies where both sun and shade leaves contribute to photosynthetic carbon assimilation and productivity. Shaded leaves are estimated to contribute about 50% of total canopy carbon gain and therefore the efficiency with which shade leaves use light is a critical factor determining crop yield potential (Baker *et al.*, 1988; Long, 1993; Hikosaka *et al.*, 2016). This proportion will increase as planting densities increase. In high light, the rate of leaf CO_2_ uptake (*A*) is limited by capacity for carboxylation and regeneration of the acceptor molecule for CO_2_. In low light, however, *A* is primarily dependent on the ability of the leaf to capture light and convert it with maximum efficiency towards carbon assimilation.

Photosynthetic efficiency under limiting light is defined by the apparent maximum quantum yield of CO_2_ assimilation $(\Phi_{{\text{CO}}_{\text{2}}\text{max,app}}),$ measured as the initial slope of the response of *A* to incident photon flux (*Q*). $\Phi_{{\text{CO}}_{\text{2}}\text{max,app}}$ is the product of leaf fractional light absorptance (α) and the intrinsic maximum efficiency with which the leaf can transduce absorbed photons into net CO_2_ assimilation, i.e. the absolute maximum quantum yield of CO_2_ assimilation $\left(\Phi_{{\text{CO}}_{\text{2}}\text{max,abs}}\right)$ (Long *et al.*, 1993). $\Phi_{{\text{CO}}_{\text{2}}\text{max,app}}$ is the key determinant of efficiency of leaf photosynthesis under light-limiting conditions (Baker *et al.*, 1988; Long, 1993; Long and Hällgren, 1993; Long *et al.*, 1993; Singsaas *et al.*, 2001; Long *et al.*, 2006).

In the classical studies of shade adaptation in C_3_ plants, it was found that $\Phi_{{\text{CO}}_{\text{2}}\text{max,app}}$ was maintained or increased in shade adapted leaves, maximizing the use of light in the shade. At the same time capacity for light-saturated photosynthesis (*A*_sat_) declined, reflecting in particular a decrease in Rubisco content (Björkman, 1981; Givnish, 1988). As canopies develop, this appears a component of a broad acclimation strategy in which various leaf traits are adjusted to optimize resource use with increasing shade (Niinemets *et al.*, 2015).

$\Phi_{{\text{CO}}_{\text{2}}\text{max,app}}$ was constant in all green leaves irrespective of leaf position and canopy depth in two independent studies of photosynthesis in field stands of modern cultivars of wheat (*Triticum aestivum* L.) (Beyschlag *et al.*, 1990; Hoyaux *et al.*, 2008). Similarly, $\Phi_{{\text{CO}}_{\text{2}}\text{max,app}}$ did not vary with depth into the canopy in wild oats (*Avena fatua* L.) growing in a wheat crop (Beyschlag *et al.*, 1990), and did not vary in grapevine (*Vitis vinifera* L.) leaves throughout the canopy (Cartechini and Palliotti, 1995). Therefore, it appears that $\Phi_{{\text{CO}}_{\text{2}}\text{max,app}}$ is maintained as expected in the lower canopy of these field-grown C_3_ crops. However, a study of perennial forage grasses showed much greater reductions in photosynthesis and productivity in C_4_ species relative to their C_3_ counterparts upon shading in the field, suggesting a possible difference between the two photosynthetic types in their ability to acclimate to shaded field conditions (Kephart *et al.*, 1992). In today’s intensive cultivation, C_4_ crops are grown at high population densities leading to leaf area indices (LAI), i.e. layers of leaves per unit ground area, of up to 6 (Dohleman and Long, 2009; Tian *et al.*, 2011; Srinivasan *et al.*, 2016). Continued development of germplasm capable of planting at still higher densities will likely lead to even higher LAI and more shaded layers (Li *et al.*, 2015). It is therefore critical to know whether key C_4_ crops are capable of maintaining $\Phi_{{\text{CO}}_{\text{2}}\text{max,app}}$ as leaves become progressively shaded in the field with canopy development, as in the classical studies of shade acclimation in C_3_ species.

While studies of $\Phi_{{\text{CO}}_{\text{2}}\text{max,app}}$ and $\Phi_{{\text{CO}}_{\text{2}}\text{max,abs}}$ span a wide variety of species and environments (Björkman and Demmig, 1987; Long *et al.*, 1993), none have focused on field stands of C_4_ crops grown under the high density populations of modern cultivation. In a natural environment, the C_4_ understory shrub *Euphorbia forbesii* Sherff. maintained a high $\Phi_{{\text{CO}}_{\text{2}}\text{max,app}}$ in a forest understory (Pearcy and Calkin, 1983). Here, however, the leaves develop in the shade while in canopies of maize (*Z. mays* L.) and other C_4_ crop stands leaves develop in full sunlight and are then shaded by younger leaves. In general, less is known about how light-limited photosynthesis acclimates in crop canopies in the field, even though other aspects of shade acclimation such as specific leaf area, light-saturated photosynthetic capacity and nitrogen content have been examined extensively in forests and some crop stands (Anten *et al.*, 1995, 1998; Brooks *et al.*, 1996; Drouet and Bonhomme, 1999; Niinemets *et al.*, 2015; Niinemets, 2016*a*,*b*).

In prior studies of $\Phi_{{\text{CO}}_{\text{2}}\text{max,app}}$ and $\Phi_{{\text{CO}}_{\text{2}}\text{max,abs}}$ in C_4_ plants, ‘shade’ treatments have typically been obtained by growing plants at low light levels or shading them with neutral density shade cloth (Ludlow and Wilson, 1971; Ehleringer and Pearcy, 1983; Pearcy and Franceschi, 1986; Tazoe *et al.*, 2008). This likely oversimplifies the shade conditions present in field canopies, where reduced light quantity is accompanied by changes in light quality, wind, humidity and temperature (McCree, 1972; Burkey and Wells, 1991; Niinemets and Valladares, 2004; Gutschick, 2016). Most notably, shade cloth fails to mimic the declines in blue and in red to far red ratio, both of which are now known to be critical to several developmental processes (Chen *et al.*, 2004).

With the perceived need to increase crop production, given forecasts of future demand (Long *et al.*, 2015*b*), it becomes increasingly important to understand leaf photosynthetic shade response of major C_4_ crops and in turn whether this could affect canopy photosynthesis and productivity (Miguez *et al.*, 2009; Zhu *et al.*, 2010; Yin and Struik, 2012, 2015). Maize (*Z. mays* L.) is the largest single primary foodstuff produced globally, with one-third of that production in the US cornbelt (FAOSTAT, 2016; USDA-NASS, 2016). *Miscanthus* × *giganteus* Greef et Deu.) is one of the most productive second generation bioenergy crops (Clifton-Brown *et al.*, 2004; Arundale *et al.*, 2014*a*; Heaton *et al.*, 2008, 2010). These important crops were chosen for this study to represent established or emerging agricultural systems, examined near the center of their US areas of production, where some of the highest yields of both crops have been reported (Dohleman and Long, 2009; Long *et al.*, 2015*a*). They are members of the grass tribe Andropogonae and closely related to the two other major C_4_ crops based on global production: sorghum (*Sorghum bicolor* (Lu.) Moench) and sugarcane (*Saccharum officinarum* L.). All members of this tribe belong to the same clade of C_4_ evolution and are classified as ‘NADP-ME type’ (Sage *et al.*, 2011).

The hypothesis that $\Phi_{{\text{CO}}_{\text{2}}\text{max,app}}$ and $\Phi_{{\text{CO}}_{\text{2}}\text{max,abs}}$ are maintained or increased in lower canopy leaves of these crops, as anticipated from the shade response observed in C_3_ species, was tested. Leaf gas-exchange measurements combined with measurements of absorptance were used to determine $\Phi_{{\text{CO}}_{\text{2}}\text{max,app}}$, α and $\Phi_{{\text{CO}}_{\text{2}}\text{max,abs}}$ in upper and lower canopy leaves of field stands of *M.* × *giganteus* and *Z. mays*. Measured values of photosynthetic parameters were then integrated into a crop canopy model to determine the effect of shade acclimation on total crop carbon assimilation.

## Materials and methods

### Plant material

Plants were sampled from mature replicated stands of *M.* × *giganteus* and *Z. mays* on the farm of the University of Illinois Agricultural Research Station near Champaign, IL, USA (40°02′N, 88°14′W, 228 m above sea level) in two consecutive growing seasons. Soils at this site are deep Drummer/Flanagan series (a fine silty, mixed, mesic Typic Endoaquoll) with high organic matter typical of the central Illinois region of the Corn Belt. Established, unfertilized field plots of the ‘Illinois’ clone of *M.* × *giganteus* were used, as described previously (Dohleman and Long, 2009; Dohleman *et al.*, 2012; Arundale *et al.*, 2014*a*,*b*). On adjacent plots, a high-yielding modern *Z. mays* hybrid, cv. Dekalb DK61-69, was planted, once soil temperature exceeded 10 °C. Both crops were rainfed and the *Z. mays* received standard fertilization of 180 kg [N] ha^–1^, prior to planting, in line with regional production practice. Once the canopy of each crop had closed (*ca*. LAI>3) measurements began and were spread across the growing season, ceasing with the beginning of senescence of the *Z. mays* crop. Achieved plant density, also in line with current agronomic practice, was approximately 8 plants m^–2^ for *Z. mays* (Dohleman and Long, 2009). The original stands of *M.* × *giganteus* were planted at 1 plant m^–2^, but tillering resulted in a stem density of approximately 100 tillers m^–2^ in subsequent years (Heaton *et al.*, 2008). This led to an LAI during this period of ~4 in plots for *Z. mays* and 4–6 for *M.* × *giganteus* (Dohleman *et al.*, 2009). To allow transfer to the laboratory for photosynthetic analysis, stems of each species were cut at the base before dawn, the cut ends immersed in water and immediately recut to avoid any air blockage in the xylem. This avoided possible effects of photoprotection or transient water stress that might develop over the course of the day. Prior use of this technique has shown that detached shoots of both crops maintain photosynthetic rates at least equal to that of field plants for 24 hours after cutting (Leakey *et al.*, 2006; Dohleman *et al.*, 2009).

To isolate the effect of age on *M.* × *giganteus* leaves, in a separate experiment, six plants were grown in a soil-free medium (LC1, Sungro Horticulture, Agawam, MA, USA) in 23-liter pots in a controlled environment greenhouse, maintained at 25–30 °C. Pots were kept well-watered and fertilized once per week with a 20:20:20 N:P:K commercial fertilizer (Peter’s Professional; The Scotts Co., Marysville, OH, USA), applied at the manufacturer’s recommended rate. High pressure sodium lamps ensured a minimum *Q* of 300 μmol m^–2^ s^–1^ and a 14 h day length. Leaves were tagged on emergence of the ligule, and as other leaves formed above, these were artificially held to the side to avoid any shading of the tagged leaves over the next 60 d.

### Canopy light profile

The fraction of *Q* intercepted by the canopy was measured from late June to mid-August by simultaneously measuring *Q* above the mature crop canopy with a point quantum sensor (Model LI-190; LI-COR, Inc., Lincoln, NE, USA) and with a line quantum sensor (Ceptometer, Model PAR-80, Decagon Devices, Inc., Pullman, WA, USA) within the canopy. The line sensor was lowered from the top of the canopy to the base in 10 cm steps, and the proportion of incident *Q* remaining was calculated. These measurements were made between 10.00 h and 14.00 h on clear sky days when incident *Q* was ≥1400 μmol m^–2^ s^–1^.

### Photosynthesis measurements

On a single tiller of each plant of *M.* × *giganteus* or the sole stem of each *Z. mays* plant, the lowest fully green leaf and the highest fully developed leaf, as indicated by ligule emergence, were selected for measurement. Leaf CO_2_ and water vapor exchange were measured in cuvettes with controlled temperature, humidity and photon flux within a portable open gas-exchange system incorporating infra-red CO_2_ and water vapor analysers, and a modulated chlorophyll fluorimeter (LI 6400 and LI 6400–40; LI-COR, Inc.).

Leaves of both species were placed in the cuvette with incident *Q* set to 2000 μmol m^–2^ s^–1^, block temperature to 30 °C, [CO_2_] to 400 μmol mol^–1^ and leaf-to-air water vapor pressure deficit to 1.3 kPa. Light was provided by the integrated red (635 nm wavelength) and blue (465 nm wavelength) light-emitting diodes (LED) such that 10% of the light was blue, and the remainder red.

Leaves were allowed to acclimate (60–90 min) until *A* reached a steady state, then light response curves were determined by decreasing *Q* to progressively lower levels (2000, 1500, 1000, 500, 200, 180, 160, 140, 120, 100, 80, 60, 40, 20, and 0 μmol m^–2^ s^–1^). Leaves were allowed to acclimate to each step reduction in *Q*, as assessed by a resumption of a steady-state *A*, typically requiring 5–10 min. As a check for any hysteresis in the response of *A* to *Q*, similar measurements were made on three separate plants in reverse starting from zero and progressively increasing to *Q*=2000 µmol m^–2^ s^–1^, with acclimation of 15–30 min between changes in photon flux.

Upon acclimation to each photon flux, gas-exchange data were recorded and *A*, *g*_s_, and intercellular CO_2_ concentration (*c*_i_) calculated (von Caemmerer and Farquhar, 1981). On a subset of these, modulated fluorescence measurements were made, as in Yin *et al.* (2014), to derive operating quantum yield of PSII photochemistry (Φ_PSII_) using a multiphase flash protocol (Loriaux *et al.*, 2013). Light response curves were described by a four-parameter non-rectangular hyperbola and fit by a maximum-likelihood routine (Long and Hällgren, 1993). The four parameters are the initial slope of the response, the *y*-axis intercept, which represents dark respiration (*R*_d_), the upper asymptote (*A*_sat_), and a convexity factor (θ) describing the rate of transition between the initial slope and asymptote with respect to *Q.*

After each light curve was completed, leaf fractional light absorptance of photosynthetically active photon flux (α) was calculated and weighted for 90% red (635 nm wavelength) and 10% blue (465 nm wavelength) to match cuvette illumination. Measurements were made as in Singsaas *et al.* (2001) by placing the leaf on the entry and then exit ports of a Taylor integrating sphere with attached illuminator and measuring optics (LI-1800-12; LI-COR). The signal was processed through a fiber optic grating spectrometer (USB2000; Ocean Optics, Dunedin, FL, USA) and analysed with the spectrometer operating software (Spectrasuite; Ocean Optics). Absorbed photosynthetic photon flux density (*Q*_abs_) for the leaf in the cuvette was then calculated as *α.Q*, assuming that absorptance by non-photosynthetic pigments was negligible, as indicated by the observed spectra (Hikosaka *et al.*, 2016).

Although an estimate of $\Phi_{{\text{CO}}_{\text{2}}\text{max,app}}$ is given by fitting the hyperbola to the response of *A* to *Q*, this estimate can be affected by values of *A* above the initial slope of the response curve (Long *et al.*, 1993; Yin *et al.*, 2014). A more accurate estimate of $\Phi_{{\text{CO}}_{\text{2}}\text{max,app}}$ was obtained from linear regression of *A* against *Q* for six light levels, between *Q=*40 and 140 µmol m^–2^ s^–1^. $\Phi_{{\text{CO}}_{\text{2}}\text{max,abs}}$ was obtained from linear regression of *A* against *Q*_abs_ for these same light levels.

It has been suggested that $\Phi_{{\text{CO}}_{\text{2}}\text{max,app}}$ can be underestimated due to decline in Φ_PSII_ with increasing *Q*, even at very low light. An alternative method for calculation of $\Phi_{{\text{CO}}_{\text{2}}\text{max,app}}$ to correct for this has been proposed (Yin *et al.*, 2014). While this calculates the theoretical maximum quantum yield for CO_2_ assimilation, the observed linear response we have reported as $\Phi_{{\text{CO}}_{\text{2}}\text{max,app}}$ is the actual achieved maximum and is the value that contributes directly to canopy carbon assimilation. The response of *A* to *Q* may deviate from linearity at very low light due to increased respiration, i.e. the Kok effect, and at high light when *A* is no longer strictly light-limited. Performing a linear regression with data points deviating from linearity would produce erroneous estimates of $\Phi_{{\text{CO}}_{\text{2}}\text{max,app}}$ and $\Phi_{{\text{CO}}_{\text{2}}\text{max,abs}}$ (Yin *et al.*, 2014). To avoid this, we ensured that the relationship of *A* to *Q* was linear over the light range used (*Q=*40–140 μmol m^–2^ s^–1^) by examining the distribution of residuals and testing their normality for each of the regressions. Details of the statistical analysis of slopes is given below. For comparison of results from this method and that of Yin *et al.* (2014), maximum quantum yield of CO_2_ assimilation corrected for PSII quantum efficiency on an incident light basis $\left(\Phi_{{\text{CO}}_{\text{2}}\text{max,app,PSII}}\right)$ was calculated as in Yin *et al.* (2014) in 13 plants of *Z. mays* and 15 plants of *M.* × *giganteus* for which fluorescence data was recorded, as described above.

To distinguish the effect of leaf age from leaf light history on lower canopy photosynthetic efficiency an additional greenhouse experiment was undertaken with *M.* × *giganteus*, as described above. The above gas exchange measurements were repeated on the uppermost leaf in which the ligule had just emerged on six plants, and repeated on the same leaf 30 and 60 days later and after several leaves had formed above on the same stem.

### Statistical analysis

Data were analysed using PROC MIXED (SAS Institute Inc., Cary, NC, USA), and graphical displays made with SigmaPlot 11.0 software (Systat Software Inc., San Jose, CA, USA). A randomized complete block mixed model ANOVA was performed on field data to analyse the fixed effect of canopy position (*C*_i_) and species (*S*_j_) as well as their fixed interaction (*CS*_ij_), while blocking by the random main effect of year (*T*_k_). Here ε_ijk_ represents a random error term for the model. This analysis was performed on all photosynthetic parameters of interest, with *Y*_ijk_ corresponding to *A*_sat_, _$\Phi_{{\text{CO}}_{\text{2}}\text{max,abs}}$_, $\Phi_{{\text{CO}}_{\text{2}}\text{max,app}},$*$\Phi_{{\text{CO}}_{\text{2}}\text{max,app,PSII}},$R*_d_, or α.

$$Y_{\text{ijk}}=C_{\text{i}}+S_{\text{j}}+CS_{\text{ij}}+T_{\text{k}}+\varepsilon_{\text{ijk}}$$

PROC UNIVARIATE (SAS Institute Inc.) was used to verify normality of the ANOVA residuals using the Shapiro–Wilk test, with a 1% threshold probability of committing a type 1 error. Because measurements from the lower canopy were inherently more variable than from the upper, and variances differed between species, homogeneity of variance could not be assumed. Therefore, the repeated measures option of PROC MIXED was used to allow variance to differ between canopy levels and between species. When analysing $\Phi_{{\text{CO}}_{\text{2}}\text{max,app}}$ and $\Phi_{{\text{CO}}_{\text{2}}\text{max,abs}}$ least squares were weighted by the inverse of the variance of each slope calculation; this was to incorporate variability of each regression into the overall statistical model. An upper and a lower canopy leaf were measured on each of 49 plants, leading to 40 and 53 complete *A*–*Q* curves measured in *Z. mays* and *M.* × *giganteus*, respectively. Deviation from linearity in the initial slopes of the regressions of *A* against *Q*, from which $\Phi_{{\text{CO}}_{\text{2}}\text{max,app}}$ and $\Phi_{{\text{CO}}_{\text{2}}\text{max,abs}}$ were derived, was tested with PROC UNIVARIATE. This was used to verify normality of the residuals from each regression using the Shapiro–Wilk test, with a 1% threshold probability of committing a type 1 error.

A separate analysis was performed on data from the greenhouse experiment, where the effect of leaf age was isolated from that of shading as described above. A repeated measures fixed model ANOVA was performed, blocking by day of measurement and using *post hoc* Tukey’s HSD contrast statements to analyse the linear fixed effect of time on $\Phi_{{\text{CO}}_{\text{2}}\text{max,app}}$ and $\Phi_{{\text{CO}}_{\text{2}}\text{max,abs}}.$

### Modeling canopy assimilation

The function CanA of the BioCro R package (Miguez *et al.*, 2009) was modified to simulate an exponential decrease in photosynthetic parameters with cumulative leaf area from the top to the base of the canopy. The *M.* × *giganteus* canopy was divided into 10 layers containing equal fractions of LAI. For each layer, sunlit-shaded leaf areas and direct and diffuse light fluxes were calculated hourly throughout the day. Light within each canopy layer was used to calculate the rate of photosynthesis of both sunlit and shaded leaves with a coupled steady-state biochemical and stomatal model (Collatz *et al.*, 1992). Rates were then integrated through the canopy to compute hourly rates of CO_2_ assimilation per square meter of ground area, as described previously (Miguez *et al.*, 2009). Solar radiation, temperature, relative humidity, and wind speed were compiled from the nearest Surface Radiation Network (SURFRAD) site (40.05N, –88.37W) for 2012.

In the steady-state biochemical model of C_4_ photosynthesis used in BioCro, *A*_sat_ is determined by capacity for phosphoenol pyruvate (PEP) carboxylation (*V*_pmax_) at low *c*_i_ and by capacity for PEP regeneration (*V*_max_) at moderate *c*_i_. Since previous studies of both crops have shown *A*_sat_ to be determined entirely by *V*_max_ under field conditions, except during severe drought (Dohleman *et al.*, 2009; Leakey *et al.*, 2004, 2006), *V*_max_ was assumed equivalent to *A*_sat_+*R*_d_. The exponential decline of photosynthesis parameters (*V*_max_, *R*_d_, and *$\Phi_{{\text{CO}}_{\text{2}}\text{max,app}})$* was simulated after setting values at the top and bottom of the canopy to those measured in this field study, using an extinction coefficient per LAI layer (*K*=0.1) to vary the parameters between the two measured points. Selection of *K* was based on the observed decline in leaf N, as a proxy of photosynthetic capacity, measured previously in this *M.* × *giganteus* crop (Wang *et al.*, 2012). Simulations were performed for four scenarios: (1) *V*_max_ and $\Phi_{{\text{CO}}_{\text{2}}\text{max,app}}$ are held constant throughout the canopy, at the value measured in the upper canopy, (2) *V*_max_ is held constant at the value measured in the upper canopy while $\Phi_{{\text{CO}}_{\text{2}}\text{max,app}}$ decreases from top to bottom of the canopy, (3) *V*_max_ decreases from top to bottom of the canopy while $\Phi_{{\text{CO}}_{\text{2}}\text{max,app}}$ is held constant at the value measured in the upper canopy, and (4) *V*_max_ and $\Phi_{{\text{CO}}_{\text{2}}\text{max,app}}$ both decrease, as observed in the crop, from top to bottom of the canopy. In all scenarios, *R*_d_ decreases exponentially with cumulative leaf area from top to bottom of the canopy.

For the purposes of quantifying possible losses due to decline in capacity for light-limited and light-saturated photosynthesis with depth into the canopy, a leaf area index (LAI) of 5.0 was assumed for the simulation across the month of June (Dohleman and Long, 2009). Other than the changes noted above, all equations and parameters for simulating *M.* × *giganteus* canopy photosynthesis were as detailed in full previously (Miguez *et al.*, 2009).

## Results

**Fig. 1.**
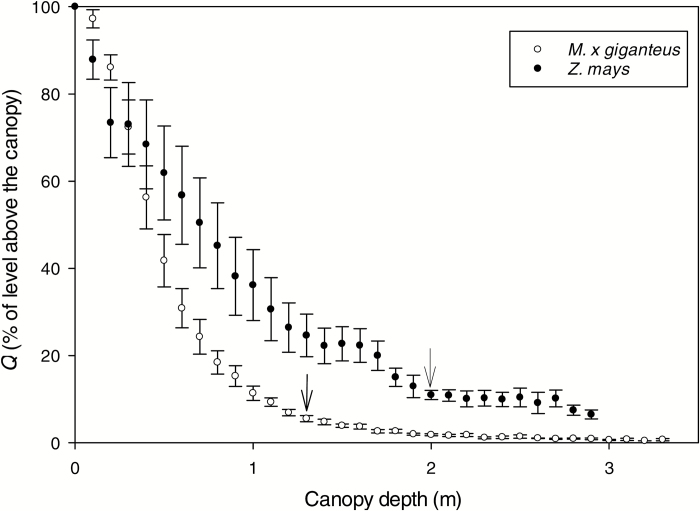
Photon flux (*Q*), as a proportion of that at the upper surface of the canopy, plotted against depth into the canopies of the field stands of *Miscanthus × giganteus* Greef et Deu. and *Zea mays* L. Measurements were made between 10.00 h and 14.00 h on clear sky days from July to August. On the *x*-axis, 0 indicates the upper surface of the canopy. Each point is the mean (±1 SE) of eight independent measurements taken at a given depth from the canopy surface. Arrows indicate approximate canopy depths where lower canopy leaves were selected from both species: 1.3 m for *M.* × *giganteus* and 2 m for *Z. mays*; these corresponded to an overlying LAI of 5.8 and 4.4, respectively. Leaves referred to as upper canopy (full sunlight) were those at the surface (canopy depth=0) and those referred to as lower canopy are indicated by arrows, where photon flux was reduced by about 90%.

Light level declined exponentially with depth into the canopy, most markedly in *M.* × *giganteus* (Fig. 1). The lowest fully green leaf was approximately 1.3 m below the canopy surface in the stands of *M.* × *giganteus* and 2 m in *Z. mays*. At those canopy levels, the measured photosynthetic photon flux density (*Q*) was 5–10% of that at the canopy surface (Fig.1). This corresponds to an overlying leaf area of between 4.4 and 5.8 m^2^.

**Fig. 2.**
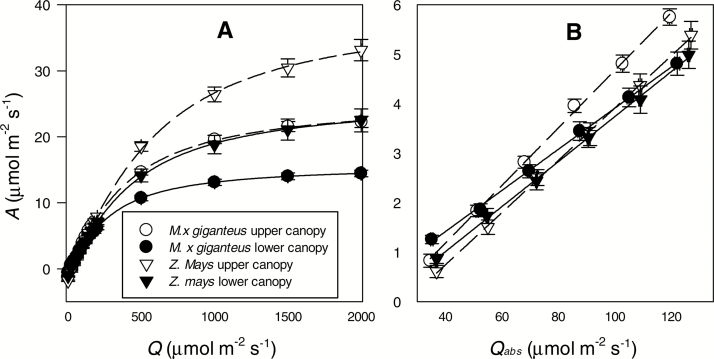
(A) Response of net CO_2_ assimilation (*A*) to incident photon flux (*Q*). (B) Strictly light limiting phase of the response of *A* to leaf absorbed photon flux (*Q*_abs_), corresponding to *Q=*40–140 μmol m^–2^ s^–1^. Results correspond to the upper and lower canopy of *Miscanthus* × *giganteus* Greef et Deu. and *Zea mays* L. at the positions indicated by Fig. 1. Open symbols represent the measured mean (±1 SE) at a given photon flux for upper canopy leaves and closed symbols lower canopy leaves. Replicate numbers of plants are as given in Table 2. Lines are the best-fit regressions to the original data points. Dashed lines represent upper canopy leaves, and solid lines lower canopy leaves.

Leaf fractional light absorptance (α) was significantly and 3% greater in lower compared with upper canopy leaves of *M.* × *giganteus*, but not different between canopy levels in *Z. mays* (Tables 1 and 2). By comparison with upper canopy leaves, values for $\Phi_{{\text{CO}}_{\text{2}}\text{max,abs}}$ and $\Phi_{{\text{CO}}_{\text{2}}\text{max,app}}$ in the lower canopy were significantly decreased by 27–29% in *M.* × *giganteus* and by 14–15% in *Z. mays*, (Tables 1 and 2, and Fig. 2B). This reduction was also apparent when the *A*–*Q* response was determined by increasing, rather than decreasing, *Q*, and when determined with adjustment for decline in PSII quantum efficiency (*P*<0.05) (Tables 1 and 2, and Supplementary Fig. S1 at *JXB* online). Residuals of each regression used to calculate $\Phi_{{\text{CO}}_{\text{2}}\text{max,app}}$ and $\Phi_{{\text{CO}}_{\text{2}}\text{max,abs}}$ were normally distributed, and therefore they were randomly distributed around *Q* and *Q*_abs_, respectively (Supplementary Figs S2–S5). This indicates *A* was linearly related to *Q* from *Q*=40 to 140 μmol m^–2^ s^–1^ and that the quantum yields measured did represent the true maxima achieved by the measured leaves.

**Table 1.** *The significance of differences in light-saturated net leaf CO*_*2*_ *uptake* (A_*sat*_*), maximum quantum yield of CO*_*2*_*assimilation on an absorbed light basis $(\Phi_{CO_{2}max,abs}),$ maximum quantum yield of CO*_*2*_*assimilation on an incident light basis $(\Phi_{CO_{2}max,app}),$ maximum quantum yield of CO*_*2*_*assimilation corrected for* PSII *quantum efficiency on an incident light basis $(\Phi_{CO_{2}max,app,PSII}),$ leaf dark respiration* (R_*d*_*), and leaf fractional light absorptance* (α*) between upper and lower canopy leaves of* Miscanthus × giganteus *Greef et Deu. and* Zea mays *L.* Values in the table are *F*-statistics. Significant differences are indicated at *P*<0.1 by #; at *P*<0.05 by *, at *P*<0.001 by **, and at *P*<0.0001 by ***.

Effect	*A*_sat_	$\Phi_{CO_{2}max,abs}$	$\Phi_{CO_{2}max,app}$	$\Phi_{CO_{2}max,app,PSII}$	*R*_d_	*α*
Canopy position main effect	74***	9.1*	7.15*	16.94***	67***	5.0*
Canopy position × species interaction	1.3	9.63*	8.73*	2.28	2.8#	8.8*

**Table 2.** *Mean values and standard error of light-saturated net leaf CO*_*2*_ *uptake* (A_*sat*_*), maximum quantum yield of CO*_*2*_*assimilation on an absorbed light basis $(\Phi_{CO_{2}max,abs}),$ maximum quantum yield of CO*_*2*_*assimilation on an incident light basis $(\Phi_{CO_{2}max,app}),$ maximum quantum yield of CO*_*2*_*assimilation corrected for PSII quantum efficiency on an incident light basis $(\Phi_{CO_{2}max,app,PSII}),$ leaf dark respiration* (R_*d*_*), and leaf fractional light absorptance* (α*) for upper and lower canopy leaves of* Miscanthus × giganteus *Greef et Deu. and* Zea mays *L.* Results are from the canopy positions indicated in Fig. 1. Statistically significant difference (Student’s *t* test) between upper and lower canopy for each species at *P*<0.1 is indicated by #; at *P*<0.05 by *, at *P*<0.001 by **, and at *P*<0.0001 by ***: in the case of a significant difference the higher of the pair is written in bold.

*M.* × *giganteus*	*A*_sat_ (*n*=24–27)		$\Phi_{CO_{2}max,abs}$ (*n*=24–27)		$\Phi_{CO_{2}max,app}$ (*n*=24–27)		$\Phi_{CO_{2}max,app,PSII}$ (*n*=15)		*R*_d_ (*n*=24–27)		α (*n*=24–27)	
	Mean	SE	Mean	SE	Mean	SE	Mean	SE	Mean	SE	Mean	SE
Upper canopy	27.6***	5.29	0.058***	0.0078	0.049***	0.0066	0.058**	0.0085	1.27***	0.39	0.851	0.021
Lower canopy	16.1	2.97	0.041	0.0097	0.039	0.0084	0.044	0.0097	0.43	0.42	0.873*	0.028
***Z. mays***	***A*_sat_** **(*n*=19–20)**		$\Phi_{CO_{2}max,abs}$ (***n*=17–20)**		$\Phi_{CO_{2}max,app}$ **(*n*=19–20)**		$\Phi_{CO_{2}max,app,PSII}$ **(*n*=13)**		***R*_d_** (***n*=19–20)**		**α** **(*n*=17–20)**	
	**Mean**	**SE**	**Mean**	**SE**	**Mean**	**SE**	**Mean**	**SE**	**Mean**	**SE**	**Mean**	**SE**
Upper canopy	42.3***	10.8	**0.053#**	0.010	0.048*	0.009	**0.054#**	0.0049	1.76***	0.36	0.906	0.016
Lower canopy	27.3	9.2	0.044	0.013	0.041	0.012	0.047	0.0134	1.21	0.43	0.904	0.012

**Fig. 3.**
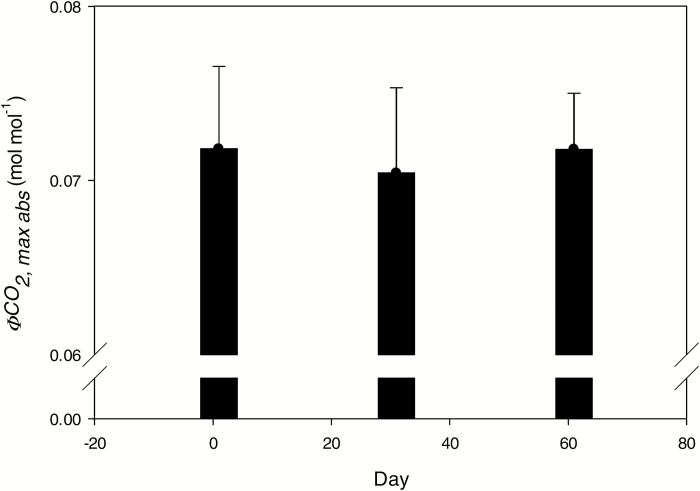
Maximum quantum yield of CO_2_ assimilation on an absorbed light basis $\left(\Phi_{{\text{CO}}_{\text{2}}\text{max,abs}}\right)$ with days after emergence of the leaf ligule in *Miscanthus* × *giganteus* Greef et Deu. Leaves were artificially maintained in unshaded conditions to separate aging from decrease in light quantity and quality, as would otherwise occur with sequential production of leaves above as a canopy develops. Each bar is the mean of six plants (±1 SE).

At higher light levels (*Q*=500 to 2000), the lower canopy of both *Z. mays* and *M.* × *giganteus* had lower photosynthetic rates than the upper canopy (Fig. 2A). This is confirmed by the significant main effect of canopy position on *A*_sat_ (Tables 1 and 2). Relative to the upper canopy leaves, lower canopy values for *A*_sat_ declined by 42% for *M.* × *giganteus* and by 35% for *Z. mays* (Table 2). Lower canopy dark respiration (*R*_d_) declined by 29% and 69% relative to the upper canopy in *M.* × *giganteus* and *Z. mays*, respectively (Tables 1 and 2). *M.* × *giganteus* leaves artificially maintained in unshaded conditions in the separate greenhouse experiment showed no significant decline in $\Phi_{{\text{CO}}_{\text{2}}\text{max,abs}}$ (*F*=1.43; *P*>0.1) or $\Phi_{{\text{CO}}_{\text{2}}\text{max,app}}$ (*F*=0.02; *P*>0.1) over 60 days (Fig. 3).

**Fig. 4.**
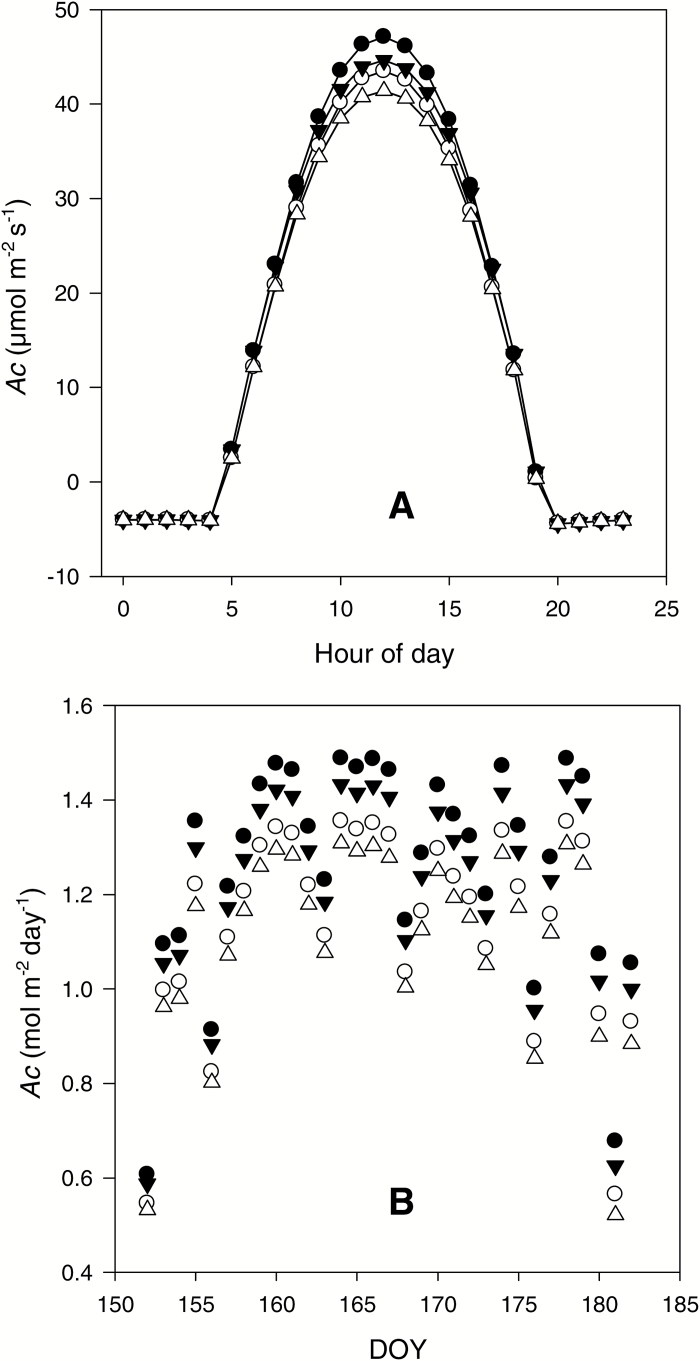
Modeled canopy CO_2_ assimilation (*A*_c_) for a *Miscanthus* × *giganteus* Greef et Deu. canopy (LAI=5) based on actual measurements of weather and canopy geometry at the site of the stands in Illinois. (A) Predicted variation of canopy CO_2_ assimilation per unit ground area (*A*_c_) across a single day (DOY 167, mid-June). This assumes for scenario 1 no decline in $\Phi_{{\text{CO}}_{\text{2}}\text{max,app}}$ or *A*_sat_ from top to bottom of the canopy (●), for scenario 2 the measured decline in $\Phi_{{\text{CO}}_{\text{2}}\text{max,app}}$ but not *A*_sat_ (○), for scenario 3 the measured decline in *A*_sat_ but not $\Phi_{{\text{CO}}_{\text{2}}\text{max,app}}$ (▼), and for scenario 4 the measured decline in both $\Phi_{{\text{CO}}_{\text{2}}\text{max,app}}$ and *A*_sat_ (∆). (B) Daily canopy assimilation per unit ground area across the entire month of June, where symbols are as in (A). The integrated total predicted for June for each scenario is given in Table 3.

Losses in total crop carbon assimilation due to the measured declines in *A*_sat_ and $\Phi_{{\text{CO}}_{\text{2}}\text{max,app}}$ with canopy depth were simulated in the BioCro mechanistic model of crop canopy photosynthesis. Scenario 1 represented the hypothetical condition of no decline in these parameters. The effect of the actual decline (scenario 1 *vs*. 4; Fig. 4A) was evident throughout the day and across the whole month (Fig. 4A, B). Integrated across the month the combined decline in *A*_sat_ and $\Phi_{{\text{CO}}_{\text{2}}\text{max,app}}$ cost 15% of potential carbon gain relative to the hypothetical situation of no decline in either parameter (scenario 1 *vs*. 4; Table 3). Maintaining *A*_sat_ as constant into the canopy, but allowing $\Phi_{{\text{CO}}_{\text{2}}\text{max,app}}$ alone to decline as observed, resulted in a 4% increase in canopy carbon gain (scenario 2 *vs*. 4). Maintaining $\Phi_{{\text{CO}}_{\text{2}}\text{max,app}}$ at the upper canopy value into the lower canopy, but allowing *A*_sat_ to decline as observed, resulted in a 10% increase in canopy carbon gain (scenario 3 *vs*. 4; Table 3).

**Table 3.** *Modeled canopy CO*_*2*_ *assimilation* (A_c_*) integrated over the month of June for a* Miscanthus × giganteus *Greef et Deu. canopy (LAI=5) based on actual measurements of weather and canopy geometry at the site of the stands in Illinois* This assumes for scenario 1 no decline in $\Phi_{{\text{CO}}_{\text{2}}\text{max,app}}$ or *A*_sat_ from top to bottom of the canopy, for scenario 2 the measured decline in $\Phi_{{\text{CO}}_{\text{2}}\text{max,app}}$ but not *A*_sat_, for scenario 3 the measured decline in *A*_sat_ but not $\Phi_{{\text{CO}}_{\text{2}}\text{max,app}},$ and for scenario 4 the measured decline in both $\Phi_{{\text{CO}}_{\text{2}}\text{max,app}}$ and *A*_sat_. n.a., not applicable.

	Scenario			
	1	2	3	4
*A*_c_ (mol m^–2^)	39.1	35.3	37.5	34.0
Increase in *A*_c_ over scenario 4 (%)	15%	4%	10%	n.a.

## Discussion

In contrast to findings of the classical studies of shade acclimation, the maximum quantum yield of leaves showed a significant decline under the shade conditions of the lower canopy of these two C_4_ crops. The observation that the absolute and apparent maximum quantum yield of CO_2_ assimilation $(\Phi_{{\text{CO}}_{\text{2}}\text{max,abs}}$ and $\Phi_{{\text{CO}}_{\text{2}}\text{max,app}})$ both decline in field stands of these highly productive C_4_ crops appears new and surprising. Even when quantum yield was adjusted for decline of PSII quantum efficiency at low light (Yin *et al.*, 2014), $\Phi_{{\text{CO}}_{\text{2}}\text{max,app,PSII}}$ was significantly reduced in the lower canopy (Tables 1 and 2, and Fig. 2B). However, for the purposes of this study the decline in $\Phi_{{\text{CO}}_{\text{2}}\text{max,app}}$ is the important measure, since based solely on CO_2_ assimilation it provides an unequivocal measure of the actual efficiency with which carbon is assimilated in low light. This suggests large losses of potential crop carbon uptake could be avoided if $\Phi_{{\text{CO}}_{\text{2}}\text{max,app}}$ was maintained with canopy depth (Table 3 and Fig. 4).

Compared with the sun leaves of the upper canopy, lower canopy leaves showed several traits typical of shade acclimation: reduced *A*_sat_, reduced dark respiration (*R*_d_), and in the case of *M.* × *giganteus* a significant increase in absorptance (α) (Table 2). While these changes fit with expectations of shade acclimation (Boardman, 1977; Givnish, 1988; Björkman, 1981), loss of 14–29% of efficiency of photosynthesis in low light $\left(\Phi_{{\text{CO}}_{\text{2}}\text{max,abs}}\right)$ does not. A loss of low-light photosynthetic efficiency in shaded leaves was not seen in field crops in two separate studies of wheat (Hoyaux *et al.*, 2008; Beyschlag *et al.*, 1990). This parallels studies of non-crop plants. Young upper sun and old lower shade leaves of the semi-arid arborescent C_3_ monocot *Beaucarnea stricta* Lem. and the C_3_ wet forest understory fern *Davallia bullata* Wail. ex Hook. showed identical values of $\Phi_{{\text{CO}}_{\text{2}}\text{max,abs}},$ suggesting no loss of efficiency of photosynthesis in shade, while *A*_sat_ was decreased by >70% in both species (Long *et al.*, 1993). Similarly, no decline in $\Phi_{{\text{CO}}_{\text{2}}\text{max,abs}}$ was seen between sun and shade leaves of a mangrove forest (Suwa and Akio, 2008) or in wild oat growing in a wheat canopy (Beyschlag *et al.*, 1990).

Lack of shade acclimation has been observed in C_4_ plants: in the NADP-ME monocot *Z. mays* and NAD-ME dicot *Amaranthus retroflexus* L. cultivated in controlled environment cabinets, growth in high *vs*. low light had no effect on $\Phi_{{\text{CO}}_{\text{2}}\text{max,abs}}.$ The same was seen in the NADP-ME dicot *Euphorbia forbesii* Sheriff. and the mixed NAD-ME and NADP-ME dicot *Gomphrena globosa* L. when grown in a greenhouse either in full sunlight or under a 90% shade cloth (Ehleringer and Pearcy, 1983). $\Phi_{{\text{CO}}_{\text{2}}\text{max,app}}$ was unchanged in the PCK monocot *Panicum maximum* Jacq. grown in a greenhouse either in full sunlight or under layers of shade cloth (Ludlow and Wilson, 1971). The NAD-ME dicot *Amaranthus cruentus* L. not only maintained $\Phi_{{\text{CO}}_{\text{2}}\text{max,app}}$ when grown under shade cloth, but also showed evidence of positive acclimation in terms of decreased bundle sheath leakiness (Tazoe *et al.*, 2008). Clearly there is a well-documented ability for a wide diversity of C_4_ species to maintain maximum quantum yields when growing under artificial neutral density shade. This is seen in all three major C_4_ subtypes (NADP-ME, NAD-ME, PCK) and an intermediate (NAD-ME/NADP-ME), and in both monocots and dicots, suggesting there is no inherent limitation of C_4_ photosynthesis at low light.

In contrast, the leaves that became progressively shaded as other leaves formed above them *in situ* in the current study suffered a decrease in $\Phi_{{\text{CO}}_{\text{2}}\text{max,app}}$ and $\Phi_{{\text{CO}}_{\text{2}}\text{max,abs}}.$ This has not been reported previously, but given the large numbers of leaves examined here, almost 100, it is clearly a statistically proven feature of these production stands of *Z. mays* and *M.* × *giganteus*. As noted above, maximum quantum yields of C_4_ species, including *Z. mays*, do not decline when *Q* is reduced with shade cloth. This suggests that some other feature of the lower canopy causes the loss observed under the shade of other leaves in a field setting.

Because of the development pattern of these crops, shade leaves were several weeks older than those in which the ligule had just emerged at the top of the canopy. Could age be a determining factor? In our greenhouse study of *M.* × *giganteus* in which shading of leaves was prevented as new leaves were formed above them, there was no loss of $\Phi_{{\text{CO}}_{\text{2}}\text{max,abs}},$ even at 60 days. This indicates that the loss is not due to age or leaf position on the stem, but rather a direct response to shading by other leaves or canopy position (Fig. 3). $\Phi_{{\text{CO}}_{\text{2}}\text{max,abs}}$ measured in the greenhouse was generally greater than in the field, possibly because the greenhouse has slightly lower light than the outside and the environment is more constant and more humid (Table 2 and Fig. 3). This may help avoid cumulative damage that can accrue in the harsher and more variable field environment, for example following cooler mornings coupled with high light exposure (Baker *et al.*, 1989; Farage *et al.*, 2006). Clearly, this manipulation needs to be attempted in field conditions, but at a minimum this experiment demonstrates that the loss is not due to chronological age. Notably, the $\Phi_{{\text{CO}}_{\text{2}}\text{max,abs}}$ observed in this protected environment of 0.072 mol mol^–1^ (Fig. 3) is almost identical to $\Phi_{{\text{CO}}_{\text{2}}\text{max,app,PSII}}$ measured for *Z. mays* grown under similar controlled greenhouse conditions (Yin *et al.*, 2011, 2014).

Although *A*_sat_ and $\Phi_{{\text{CO}}_{\text{2}}\text{max,abs}}$ will decline in the lower leaves of plant canopies at the onset of leaf senescence (Ono *et al.*, 2001; Niinemets *et al.*, 2015; Niinemets, 2016*a*,*b*; Pons, 2016), the high values for leaf fractional light absorptance (α) indicate that leaves measured here in the lower canopy were still healthy and not senescent when they were measured. Relative to the upper canopy, α of lower canopy leaves was maintained in *Z. mays*, and significantly increased in *M.* × *giganteus* (Table 2). This increase was small, but this is not surprising given that α in the upper canopy was already high and close to the maximum reported for healthy green leaves across a range of species (Long *et al.*, 1993).

Decline in *A*_sat_ as leaves in canopies become shaded is commonly associated with the nitrogen economy of the plant, i.e. remobilizing nitrogen from major sinks, notably Rubisco, to provide nitrogen to upper canopy leaves (Evans, 1993; Osborne *et al.*, 1998; Niinemets *et al.*, 2015; Niinemets, 2016*b*). This is seen in both C_3_ and C_4_ canopies (Anten *et al.*, 1995). However, theoretical analysis of the proteins lost in this process suggested that this remobilization, while lowering *A*_sat_, should not lower $\Phi_{{\text{CO}}_{\text{2}}\text{max,abs}}$ (Hikosaka and Terashima, 1995).

Generally, measurements were more variable in the lower canopy compared with the upper canopy, and in *Z. mays* compared with *M.* × *giganteus*, although these differences were small (Table 2). Greater variability of the lower canopy could be explained by variation of leaf insertion height throughout the duration of the experiment, where leaves measured in the middle of the growing season, at peak LAI, were exposed to lower light intensities. *Z. mays* transitioned from vegetative to reproductive growth during the course of these measurements, while *M.* × *giganteus* remained in the vegetative phase.

Another possible cause of decreased $\Phi_{{\text{CO}}_{\text{2}}\text{max,app}}$ and $\Phi_{{\text{CO}}_{\text{2}}\text{max,abs}}$ is the altered light quality of the lower canopy. Light here is enriched in green and far-red relative to red and blue. Our measurements were made with a single spectral distribution of light based on blue and red LEDs. Although chlorophyll absorbs most strongly in the blue and red, at the high chlorophyll concentrations of healthy leaves, there is little difference in the absorptivity of green and red light or in their direct effect on quantum efficiency of CO_2_ assimilation (McCree, 1972). However, altered light quality, in particular the ratio of red to far red light, is known to play a major role in phytochrome mediated shade avoidance responses of plants (Casal, 2013; Pons, 2016). Far-red to red ratios are increased about four-fold in *T. aestivum* and *Z. mays* canopies at the depth at which 80% of total light has been intercepted (Sattin *et al.*, 1994). While this would not be represented when shade is simulated by growing plants in reduced light or under neutral density shade cloth, as in studies described previously, plants growing in a forest understory do experience this altered light composition. *In situ* measurements of the understory shrub *E. forbesii* gave a high $\Phi_{{\text{CO}}_{\text{2}}\text{max,app}}$ of 0.053, exceeding that of co-occurring C_3_ species and allowing them to achieve similar photosynthetic carbon gain (Pearcy and Calkin, 1983). While this shows that the decline observed here is not inherent in C_4_ photosynthesis, *E. forbesii* is taxonomically distant from the grasses used in our study and belongs to a completely independent line of C_4_ evolution (Sage *et al.* 2011). Additional experimentation would be necessary to determine whether light quality causes a decline in $\Phi_{{\text{CO}}_{\text{2}}\text{max,app}}$ and $\Phi_{{\text{CO}}_{\text{2}}\text{max,abs}}$ in C_4_ crops.

Leaves of species adapted to high light conditions may lack the plasticity to effectively acclimate to shade conditions, particularly in C_4_ plants, which show reduced plasticity in changing light environments when compared with C_3_ plants (Sage and McKown, 2006; Niinemets *et al.*, 2015; Niinemets, 2016*b*). While fast-growing grasses such as *M.* × *giganteus* are highly plastic in their remobilization of N when compared with other canopy-forming plants, this should primarily impact *A*_sat_ and not $\Phi_{{\text{CO}}_{\text{2}}\text{max,abs}}$ (Anten *et al.*, 1998; Hikosaka and Terashima, 1995; Niinemets *et al.*, 2015; Niinemets, 2016*b*). In addition, developmental effects unique to C_4_ physiology such as bundle-sheath leakage can cause negative acclimation to low light (Kromdijk *et al.*, 2008; Niinemets *et al.*, 2015).

Increasing stand density through combined efforts of breeding and agronomy has been a key factor in recent increases in yields of *Z. mays* (Duvick, 2005; Liu and Tollenaar, 2009; Li *et al.*, 2011). *M.* × *giganteus* has been selected as an emerging high production C_4_ bioenergy crop in part for its ability to be grown at high stem densities (Heaton *et al.*, 2010). These trends toward higher stem densities and LAI will result in an ever-increasing proportion of crop carbon gain contributed over a day by shaded leaves. The findings here suggest that loss of efficiency of light-limited photosynthesis with shade, may result in a diminishing rate of return with further planting density increases, unless means are found to maintain $\Phi_{{\text{CO}}_{\text{2}}\text{max,app}}$ within the canopy. The importance of shade photosynthesis is evident in our results: maintaining $\Phi_{{\text{CO}}_{\text{2}}\text{max,app}}$ from top to bottom (scenario 3) would improve canopy carbon gain (*A*_c_) more than twice as much as maintaining *A*_sat_, and without the need for additional leaf nitrogen (scenario 2, Table 3). Although decline in *A*_sat_ with shading appears almost universal and a key factor in stand nitrogen use efficiency, advances in bioengineering might soon provide means, paralleling ‘stay-green’, that prevent this decline. However, as noted above, the gain in carbon assimilation would be small compared with maintenance of $\Phi_{{\text{CO}}_{\text{2}}\text{max,app}}.$

Indirect evidence of this limitation in this clade of C_4_ grasses may come from a comparison of two productive perennial grasses. *M.* × *giganteus* is recognized as a highly productive bioenergy crop, a quality often related to its use of C_4_ photosynthesis (Heaton *et al.*, 2010). However, a paradox here is the fact that the Mediterranean C_3_ grass *Arundo donax* appears equally, if not more productive, when the two crops are grown side by side. *Arundo donax* produces an equally dense canopy, but shows a high $\Phi_{{\text{CO}}_{\text{2}}\text{max,app}},$ which may explain what has until now appeared a paradox (Webster *et al.*, 2016). Indeed, shade acclimation is of greatest importance in crops such as these, where dense canopies are formed (Niinemets, 2016*b*).

Only single genotypes of the two species were considered here. *Z. mays* is the most important crop globally in terms of total grain production and *Miscanthus* species appear the most productive of the emerging perennial C_4_ temperate bioenergy crops (Heaton *et al.*, 2010; Long *et al.*, 2015*a*). Sorghum (*Sorghum bicolor* L. Moench) and sugarcane (*Saccharum officinarum* L.) are the next most important C_4_ crops after *Z. mays* in terms of area planted and value. Both are closely related to *Miscanthus* as revealed by recent genomic analysis, and like the more distantly related *Zea* are all within the tribe Andropogoneae. They form part of a monophyletic branch of evolution of C_4_ NADP-ME genera (Swaminathan *et al.*, 2010, 2012). This close relationship suggests that the other major C_4_ crops, i.e. sorghum and sugarcane, might suffer the limitation observed here. Why could this apparent Achilles heel be present?

The ancestors of maize and *Miscanthus* appear to have existed in very open habitats, where water and nutrient deficiencies would have limited leaf area. There may therefore have been little evolutionary pressure for maintenance of photosynthetic efficiency in shade conditions. Clearly a next step will be to examine within species variability in diversity panels to identify possible breeding resources and establish the taxonomic breadth of this loss of $\Phi_{{\text{CO}}_{\text{2}}\text{max,app}}.$ If the mechanisms underlying this loss are unraveled then this may open the way to bioengineer maintenance of $\Phi_{{\text{CO}}_{\text{2}}\text{max,app}}$ with canopy depth in these crops. An up to 10% increase in the productivity of some of the world’s most important crops would seem to make this a target well worth pursuing.

It is estimated that the world may need to double production of primary foodstuffs, of which maize is the largest single component, by 2050 (Tilman and Clark, 2015). Since the approaches used in the Green Revolution are reaching their biological limits, identifying new opportunities to increase genetic yield potential will be critical (Long *et al.*, 2015*b*; Kromdijk *et al.*, 2016). Understanding the cause of the decline in $\Phi_{{\text{CO}}_{\text{2}}\text{max,app}}$ and its extent will be necessary to determine whether this apparent Achilles heel to an otherwise most important group of crops can be avoided and a substantial gain in productivity achieved.

## Supplementary Material

Supplementary Data

## Abbreviations:

α: leaf fractional light absorptance (0 to 1, dimensionless)
$\Phi_{CO_{2}max,abs}$: maximum quantum yield of CO_2_ assimilation on an absorbed light basis, i.e. maximum of δ*A*/δ*Q*_abs_ as defined by the initial slope of the response of *A* to *Q*_abs_ (mol mol^–1^)
$\Phi_{CO_{2}max,app},$: maximum quantum yield of CO_2_ assimilation on an incident light basis, i.e. maximum of δ*A/*δ*Q* as defined by the initial slope of the response of *A* to *Q* (mol mol^–1^)
$\Phi_{CO_{2}max,app,PSII},$: maximum quantum yield of CO_2_ assimilation corrected for PSII quantum efficiency on an incident light basis (mol mol^–1^)
Φ_PSII_: operating quantum yield of PSII photochemistry (mol mol^–1^)
A: net leaf CO_2_ uptake (μmol m^–2^ s^–1^)
*A*_c_: net canopy CO_2_ uptake per unit ground area (μmol m^–2^ s^–1^ or mol m^–2^ day^–1^)
*A*_sat_: light-saturated *A* (μmol m^–2^ s^–1^)
*g*_s_: stomatal conductance (μmol m^–2^ s^–1^)
*c*_i_: intercellular CO_2_ concentration (μmol mol^–1^)
LAI: leaf area index (m^2^ m^–2^)
Q: incident photosynthetic photon flux density (μmol m^–2^ s^–1^)
*Q*_abs_: absorbed photosynthetic photon flux density, i.e. α*.Q* (μmol m^–2^ s^–1^)
*R*_d_: leaf dark respiration (μmol m^–2^ s^–1^)
*V*_max_: rate of PEP regeneration, CO_2_ saturated rate of photosynthesis (μmol m^–2^ s^–1^)
*V*_pmax_: maximum rate of PEP carboxylation (μmol m^–2^ s^–1^).

## References

[CIT0001] Anten NPR Miyazawa K Hikosaka K Nagashima H Hirose T 1998 Leaf nitrogen distribution in relation to leaf age and photon flux density in dominant and subordinate plants in dense stands of a dicotyledonous herb. Oecologia 113, 314–324. 28307815 10.1007/s004420050382

[CIT0002] Anten NPR Schieving F Werger MJA 1995 Patterns of light and nitrogen distribution in relation to whole canopy carbon gain in C_3_ and C_4_ monocotyledonous and dicotyledonous species. Oecologia 101, 504–513. 28306967 10.1007/BF00329431

[CIT0003] Arundale RA Dohleman FG Heaton EA McGrath JM Voigt TB Long SP 2014 *a* Yields of *Miscanthus × giganteus* and *Panicum virgatum* decline with stand age in the Midwestern USA. Global Change Biology. Bioenergy 6, 1–13.

[CIT0004] Arundale RA Dohleman FG Voigt TB Long SP 2014 *b* Nitrogen fertilization does significantly increase yields of stands of *Miscanthus × giganteus* and *Panicum virgatum* in multiyear trials in Illinois. Bioenergy Research 7, 408–416.

[CIT0005] Baker NR Bradbury M Farage PK Ireland CR Long SP 1989 Measurements of the quantum yield of carbon assimilation and chlorophyll fluorescence for assessment of photosynthetic performance of crops in the field. Philosophical Transactions of the Royal Society of London. Series B, Biological Sciences 323, 295–308.

[CIT0006] Baker NR Long SP Ort DR 1988 Photosynthesis and temperature, with particular reference to effects on quantum yield. Symposia of the Society for Experimental Biology 42, 347–375. 3077864

[CIT0007] Beyschlag W Barnes PW Ryel R Caldwell MM Flint SD 1990 Plant competition for light analyzed with a multispecies canopy model. II. Influence of photosynthetic characteristics on mixtures of wheat and wild oat. Oecologia 82, 374–380. 28312714 10.1007/BF00317486

[CIT0008] Björkman O 1981 Responses to different quantum flux densities. In: Lange OL Nobel PS, eds. Encyclopedia of plant physiology, Vol. 12a. Physiological plant ecology I. Springer: Berlin, 57–107.

[CIT0009] Björkman O Demmig B 1987 Photon yield of O_2_ evolution and chlorophyll fluorescence characteristics at 77 K among vascular plants of diverse origins. Planta 170, 489–504. 24233012 10.1007/BF00402983

[CIT0010] Boardman NK 1977 Comparative photosynthesis of sun and shade plants. Annual Review of Plant Physiology and Plant Molecular Biology 28, 355–377.

[CIT0011] Brooks JR Sprugel DG Hinckley TM 1996 The effects of light acclimation during and after foliage expansion on photosynthesis of *Abies amabilis* foliage within the canopy. Oecologia 107, 21–32. 28307188 10.1007/BF00582231

[CIT0012] Burkey KO Wells R 1991 Response of soybean photosynthesis and chloroplast membrane function to canopy development and mutual shading. Plant Physiology 97, 245–252. 16668377 10.1104/pp.97.1.245 PMC1080990

[CIT0013] Cartechini A Palliotti A 1995 Effect of shading on vine morphology and productivity and leaf gas-exchange characteristics in grapevines in the field. American Journal of Enology and Viticulture 46, 227–234.

[CIT0014] Casal JJ 2013 Photoreceptor signaling networks in plant responses to shade. Annual Review of Plant Biology 64, 403–427. 10.1146/annurev-arplant-050312-120221 23373700

[CIT0015] Chen M Chory J Fankhauser C 2004 Light signal transduction in higher plants. Annual Review of Genetics 38, 87–117. 10.1146/annurev.genet.38.072902.092259 15568973

[CIT0016] Clifton-Brown JC Stampfl PF Jones MB 2004 Miscanthus biomass production for energy in Europe and its potential contribution to decreasing fossil fuel carbon emissions. Global Change Biology 10, 509–518.

[CIT0017] Collatz GJ Ribas-Carbo M Berry JA 1992 Coupled photosynthesis-stomatal conductance model for leaves of C_4_ plants. Australian Journal of Plant Physiology 19, 519–538.

[CIT0018] Dohleman FG Heaton EA Arundale RA Long SP 2012 Seasonal dynamics of above- and below-ground biomass and nitrogen partitioning in *Miscanthus × giganteus* and *Panicum virgatum* across three growing seasons. Global Change Biology. Bioenergy 4, 534–544.

[CIT0019] Dohleman FG Heaton EA Leakey AD Long SP 2009 Does greater leaf-level photosynthesis explain the larger solar energy conversion efficiency of Miscanthus relative to switchgrass? Plant, Cell & Environment 32, 1525–1537. 10.1111/j.1365-3040.2009.02017.x 19558624

[CIT0020] Dohleman FG Long SP 2009 More productive than maize in the Midwest: How does Miscanthus do it? Plant Physiology 150, 2104–2115. 19535474 10.1104/pp.109.139162 PMC2719137

[CIT0021] Drouet JL Bonhomme R 1999 Do variations in local leaf irradiance explain changes to leaf nitrogen within row maize canopies? Annals of Botany 84, 61–69.

[CIT0022] Duvick DN 2005 The contribution of breeding to yield advances in maize (*Zea mays L*.). Advances in Agronomy 86, 83–145.

[CIT0023] Ehleringer J Pearcy RW 1983 Variation in quantum yield for CO_2_ Uptake among C_3_ and C_4_ Plants. Plant Physiology 73, 555–559. 16663257 10.1104/pp.73.3.555 PMC1066505

[CIT0024] Evans JR 1993 Photosynthetic acclimation and nitrogen partitioning within a Lucerne canopy. 1. Canopy characteristics. Australian Journal of Plant Physiology 20, 55–67.

[CIT0025] **FAOSTAT** 2016 Database collection of the Food and Agriculture Organization of the United Nations. http://faostat3.fao.org/

[CIT0026] Farage PK Blowers D Long SP Baker NR 2006 Low growth temperatures modify the efficiency of light use by photosystem II for CO_2_ assimilation in leaves of two chilling-tolerant C_4_ species, *Cyperus longus* L. and *Miscanthus × giganteus*. Plant, Cell & Environment 29, 720–728. 10.1111/j.1365-3040.2005.01460.x 17080621

[CIT0027] Givnish TJ 1988 Adaptation to sun and shade—a whole-plant perspective. Australian Journal of Plant Physiology 15, 63–92.

[CIT0028] Gutschick VP 2016 Leaf energy balance: basics, and modeling from leaves to canopies. In: Hikosaka K Niinemets U Anten NPR, eds. Canopy photosynthesis: from basics to applications. Advances in Photosynthesis and Respiration, Vol. 42 Dordrecht: Springer, 23–58.

[CIT0029] Heaton EA Dohleman FG Long SP 2008 Meeting US biofuel goals with less land: the potential of *Miscanthus*. Global Change Biology 14, 2000–2014.

[CIT0030] Heaton EA Dohleman FG Miguez AF 2010 *Miscanthus*: a promising biomass crop. Advances in Botanical Research 56, 75–137.

[CIT0031] Hikosaka K Noguchi K Terashima I 2016 Modeling leaf gas exchange. In: Hikosaka K Niinemets U Anten NPR, eds. Canopy photosynthesis: from basics to applications. Advances in Photosynthesis and Respiration, Vol. 42 Dordrecht: Springer, 60–99.

[CIT0032] Hikosaka K Terashima I 1995 A model of the acclimation of photosynthesis in the leaves of C_3_ plants to sun and shade with respect to nitrogen use. Plant Cell & Environment 18, 605–618.

[CIT0033] Hoyaux J Moureaux C Tourneur D Bodson B Aubinet M 2008 Extrapolating gross primary productivity from leaf to canopy scale in a winter wheat crop. Agricultural and Forest Meteorology 148, 668–679.

[CIT0034] Kephart KD Buxton DR Taylor SE 1992 Growth of C_3_ and C_4_ perennial grasses under reduced irradiance. Crop Science 32, 1033–1038.

[CIT0035] Kromdijk J Glowacka K Leonelli L Gabilly ST Iwai M Niyogi KK Long SP 2016 Improving photosynthesis and crop productivity by accelerating recovery from photoprotection. Science 354, 857–861. 27856901 10.1126/science.aai8878

[CIT0036] Kromdijk J Schepers HE Albanito F Fitton N Carroll F Jones MB Finnan J Lanigan GJ Griffiths H 2008 Bundle sheath leakiness and light limitation during C_4_ leaf and canopy CO_2_ uptake. Plant Physiology 148, 2144–2155. 18971428 10.1104/pp.108.129890 PMC2593657

[CIT0037] Leakey ADB Bernacchi CJ Dohleman FG Ort DR Long SP 2004 Will photosynthesis of maize (*Zea mays*) in the US Corn Belt increase in future CO_2_ rich atmospheres? An analysis of diurnal courses of CO_2_ uptake under free-air concentration enrichment (FACE). Global Change Biology 10, 951–962.

[CIT0038] Leakey AD Uribelarrea M Ainsworth EA Naidu SL Rogers A Ort DR Long SP 2006 Photosynthesis, productivity, and yield of maize are not affected by open-air elevation of CO_2_ concentration in the absence of drought. Plant Physiology 140, 779–790. 16407441 10.1104/pp.105.073957 PMC1361343

[CIT0039] Li J Xie RZ Wang KR Ming B Guo YQ Zhang GQ Li SK 2015 Variations in maize dry matter, harvest index, and grain yield with plant density. Agronomy Journal 107, 829–834.

[CIT0040] Li Y Ma XL Wang TY 2011 Increasing maize productivity in China by planting hybrids with germplasm that responds favorably to higher planting densities. Crop Science 51, 2391–2400.

[CIT0041] Liu WD Tollenaar M 2009 Response of yield heterosis to increasing plant density in maize. Crop Science 49, 1807–1816.

[CIT0042] Long SP 1993 The significance of light-limited photosynthesis to crop canopy carbon gain and productivity—a theoretical analysis. In: Abrol YP Mohanty P Govindjee, eds. Photosynthesis—photoreactions to plant productivity. Dordrecht: Kluwer, 547–560.

[CIT0043] Long SP Hällgren J-E 1993 Measurement of CO_2_ assimilation by plants in the field and laboratory. In: Hall DO Scurlock JMO Bolhár-nordenkampf HR Leegood RC Long SP, eds. Photosynthesis and productivity in a changing environment: a field and laboratory manual. London: Chapman & Hall, 129–167.

[CIT0046] Long SP Postl WF Bolharnordenkampf HR 1993 Quantum yields for uptake of carbon-dioxide in C_3_ vascular plants of contrasting habitats and taxonomic groupings. Planta 189, 226–234.

[CIT0044] Long SP Karp A Buckeridge MS 2015 *a* Feedstocks for biofuels and bioenergy. In: Souza G, ed. Bioenergy and sustainability. Paris: SCOPE, 302–347.

[CIT0045] Long SP Marshall-Colon A Zhu XG 2015 *b* Meeting the global food demand of the future by engineering crop photosynthesis and yield potential. Cell 161, 56–66. 25815985 10.1016/j.cell.2015.03.019

[CIT0047] Long SP Zhu XG Naidu SL Ort DR 2006 Can improvement in photosynthesis increase crop yields? Plant, Cell & Environment 29, 315–330. 10.1111/j.1365-3040.2005.01493.x 17080588

[CIT0048] Loriaux SD Avenson TJ Welles JM McDermitt DK Eckles RD Riensche B Genty B 2013 Closing in on maximum yield of chlorophyll fluorescence using a single multiphase flash of sub-saturating intensity. Plant, Cell & Environment 36, 1755–1770. 10.1111/pce.12115 23586649

[CIT0049] Ludlow MM Wilson GL 1971 Photosynthesis of tropical pasture plants II. Temperature and illuminance history. Australian Journal of Biological Sciences 24, 1065–1076.

[CIT0050] McCree KJ 1972 The action spectrum, absorptance and quantum yield of photosynthesis in crop plants. Agricultural Meteorology 9, 191–216.

[CIT0051] Miguez FE Zhu X Humphries S Bollero GA Long SP 2009 A semimechanistic model predicting the growth and production of the bioenergy crop *Miscanthus × giganteus*: description, parameterization and validation. Global Change Biology. Bioenergy 1, 282–296.

[CIT0052] Niinemets U 2016 *a* Leaf age dependent changes in within-canopy variation in leaf functional traits: a meta-analysis. Journal of Plant Research 129, 313–338. 27033356 10.1007/s10265-016-0815-2 PMC5818143

[CIT0053] Niinemets U 2016 *b* Within-canopy variations in functional leaf traits: structural, chemical and ecological controls and diversity of responses. In: Hikosaka K Niinemets U Anten NPR, eds. Canopy photosynthesis: from basics to applications. Advances in Photosynthesis and Respiration, Vol. 42 Dordrecht: Springer, 101–141.

[CIT0054] Niinemets U Keenan TF Hallik L 2015 A worldwide analysis of within-canopy variations in leaf structural, chemical and physiological traits across plant functional types. New Phytologist 205, 973–993. 25318596 10.1111/nph.13096 PMC5818144

[CIT0055] Niinemets U Valladares F 2004 Photosynthetic acclimation to simultaneous and interacting environmental stresses along natural light gradients: optimality and constraints. Plant Biology 6, 254–268. 15143434 10.1055/s-2004-817881

[CIT0056] Ono K Nishi Y Watanabe A Terashima I 2001 Possible mechanisms of adaptive leaf senescence. Plant Biology 3, 234–243.

[CIT0057] Osborne CP Roche JL Garcia RL Kimball BA Wall GW Pinter PJ Morte RL Hendrey GR Long SP 1998 Does leaf position within a canopy affect acclimation of photosynthesis to elevated CO_2_? Analysis of a wheat crop under free-air CO_2_ enrichment. Plant Physiology 117, 1037–1045. 9662547 10.1104/pp.117.3.1037 PMC34920

[CIT0058] Pearcy RW Calkin HW 1983 Carbon dioxide exchange of C_3_ and C_4_ tree species in the understory of a Hawaiian forest. Oecologia 58, 26–32. 28310643 10.1007/BF00384538

[CIT0059] Pearcy RW Franceschi VR 1986 Photosynthetic characteristics and chloroplast ultrastructure of C_3_ and C_4_ tree species grown in high- and low-light environments. Photosynthesis Research 9, 317–331. 24442364 10.1007/BF00029797

[CIT0060] Pons TL 2016 Regulation of leaf traits in canopy gradients. In: Hikosaka K Niinemets U Anten NPR, eds. Canopy photosynthesis: from basics to applications. Advances in Photosynthesis and Respiration, Vol. 42 Dordrecht: Springer, 143–168.

[CIT0061] Sage RF Christin PA Edwards EJ 2011 The C_4_ plant lineages of planet Earth. Journal of Experimental Botany 62, 3155–3169. 21414957 10.1093/jxb/err048

[CIT0062] Sage RF McKown AD 2006 Is C_4_ photosynthesis less phenotypically plastic than C_3_ photosynthesis? Journal of Experimental Botany 57, 303–317. 16364950 10.1093/jxb/erj040

[CIT0063] Sattin M Zuin MC Sartorato I 1994 Light quality beneath field-grown maize, soybean and wheat canopies—red-far red variations. Physiologia Plantarum 91, 322–328.

[CIT0064] Singsaas EL Ort DR DeLucia EH 2001 Variation in measured values of photosynthetic quantum yield in ecophysiological studies. Oecologia 128, 15–23. 10.1007/s004420000624 28547085

[CIT0065] Srinivasan V Kumar P Long SP 2016 Decreasing, not increasing, leaf area will raise crop yields under global atmospheric change. Global Change Biology, DOI: 10.1111/gcb.13526. 10.1111/gcb.13526 PMC5347850 27860122

[CIT0066] Suwa R Akio H 2008 Seasonal changes in canopy photosynthesis and foliage respiration in a *Rhizophora stylosa* stand at the northern limit of its natural distribution. Wetlands Ecology and Management 16, 313–321.

[CIT0067] Swaminathan K Alabady MS Varala K 2010 Genomic and small RNA sequencing of *Miscanthus × giganteus* shows the utility of sorghum as a reference genome sequence for Andropogoneae grasses. Genome Biology 11, R12. 20128909 10.1186/gb-2010-11-2-r12 PMC2872872

[CIT0068] Swaminathan K Chae WB Mitros T 2012 A framework genetic map for *Miscanthus sinensis* from RNAseq-based markers shows recent tetraploidy. BMC Genomics 13, 142. 22524439 10.1186/1471-2164-13-142 PMC3355032

[CIT0069] Tazoe Y Hanba YT Furumoto T Noguchi K Terashima I 2008 Relationships between quantum yield for CO_2_ assimilation, activity of key enzymes and CO_2_ leakiness in *Amaranthus cruentus*, a C_4_ dicot, grown in high or low light. Plant & Cell Physiology 49, 19–29. 18032398 10.1093/pcp/pcm160

[CIT0070] Tian ZW Jing Q Dai TB Jiang D Cao WX 2011 Effects of genetic improvements on grain yield and agronomic traits of winter wheat in the Yangtze River Basin of China. Field Crops Research 124, 417–425.

[CIT0071] Tilman D Clark M 2015 Food, agriculture & the environment: can we feed the world & save the Earth? Dædalus 144, 8–23.

[CIT0072] **USDA-NASS** 2016 National Agricultural Statistics Service. www.nass.usda.gov/

[CIT0073] von Caemmerer S Farquhar GD 1981 Some relationships between the biochemistry of photosynthesis and the gas exchange of leaves. Planta 153, 376–387. 24276943 10.1007/BF00384257

[CIT0074] Wang D Maughan MW Sun JD Feng XH Miguez F Lee D Dietze MC 2012 Impact of nitrogen allocation on growth and photosynthesis of Miscanthus (*Miscanthus × giganteus*). Global Change Biology. Bioenergy 4, 688–697.

[CIT0075] Webster RJ Driever SM Kromdijk J 2016 High C_3_ photosynthetic capacity and high intrinsic water use efficiency underlies the high productivity of the bioenergy grass *Arundo donax*. Scientific Reports 6, 20694. 26860066 10.1038/srep20694 PMC4748246

[CIT0076] Yin X Belay DW van der Putten PE Struik PC 2014 Accounting for the decrease of photosystem photochemical efficiency with increasing irradiance to estimate quantum yield of leaf photosynthesis. Photosynthesis Research 122, 323–335. 25149653 10.1007/s11120-014-0030-8

[CIT0077] Yin X Struik PC 2012 Mathematical review of the energy transduction stoichiometries of C_4_ leaf photosynthesis under limiting light. Plant, Cell & Environment 35, 1299–1312. 10.1111/j.1365-3040.2012.02490.x 22321164

[CIT0078] Yin X Struik PC 2015 Constraints to the potential efficiency of converting solar radiation into phytoenergy in annual crops: from leaf biochemistry to canopy physiology and crop ecology. Journal of Experimental Botany 66, 6535–6549. 26224881 10.1093/jxb/erv371

[CIT0079] Yin X Sun Z Struik PC Gu J 2011 Evaluating a new method to estimate the rate of leaf respiration in the light by analysis of combined gas exchange and chlorophyll fluorescence measurements. Journal of Experimental Botany 62, 3489–3499. 21382918 10.1093/jxb/err038 PMC3130174

[CIT0080] Zhu XG Long SP Ort DR 2010 Improving photosynthetic efficiency for greater yield. Annual Review of Plant Biology 61, 235–261. 10.1146/annurev-arplant-042809-112206 20192734

