# Supplementary material for: Loss of photosynthetic efficiency in the shade. An Achilles heel for the dense modern stands of our most productive C4 crops?
Source: J Exp Bot. 2017 Jan 21;68(2):335–45. doi: 10.1093/jxb/erw456 (PMC5441902; doi:10.1093/jxb/erw456)
Supplement: Supplementary Data [file erw456_Supplementary_Data.zip › supplementary_figures_S1_S5.pdf]

Loss of photosynthetic efficiency in the shade. An Achilles heel for the dense modern stands of our most productive C_4 crops?

Charles P. Pignon, Deepak Jaiswal, Justin M. McGrath and Stephen P. Long

Supplementary figures

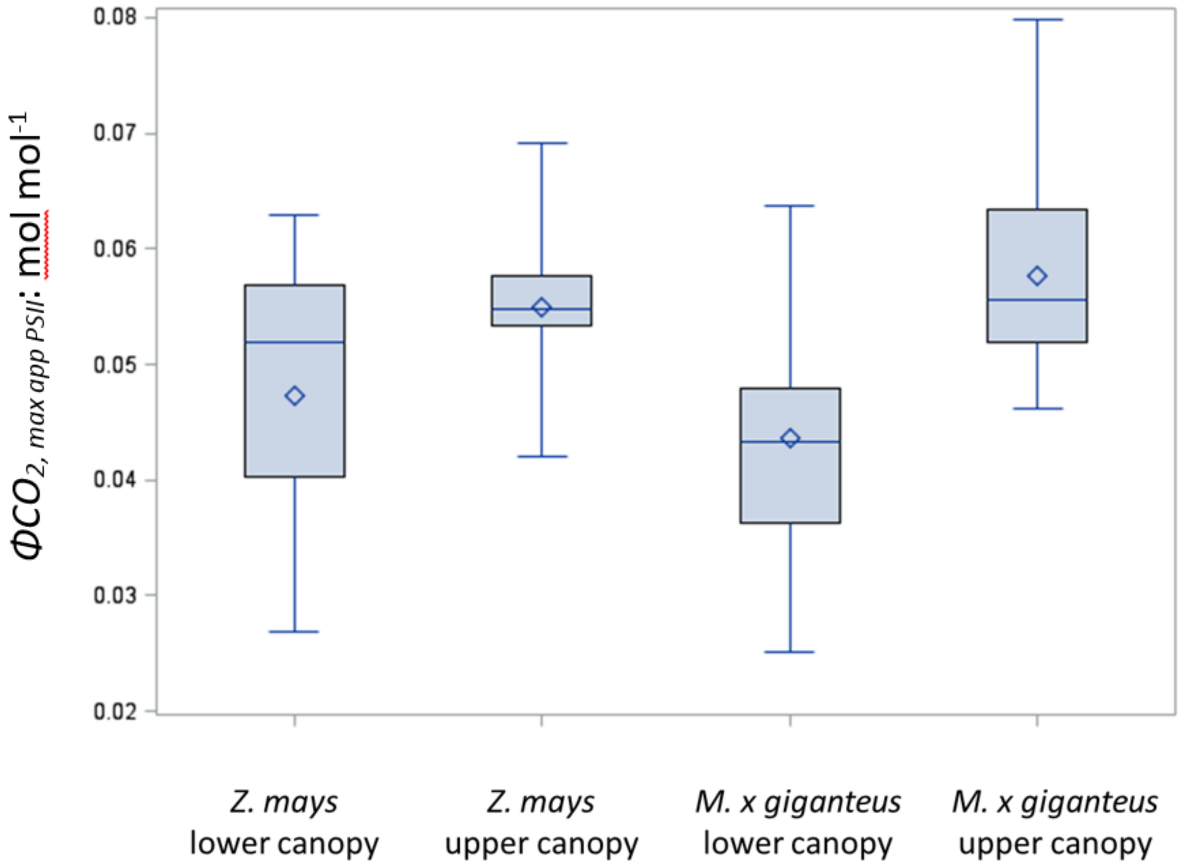

Fig. S1: Box plot of the maximum quantum yield of CO_2 assimilation on an incident light basis, calculated as in Yin et al., (2014) on measurements where fluorescence data was available (n=14-15 per species and canopy position)

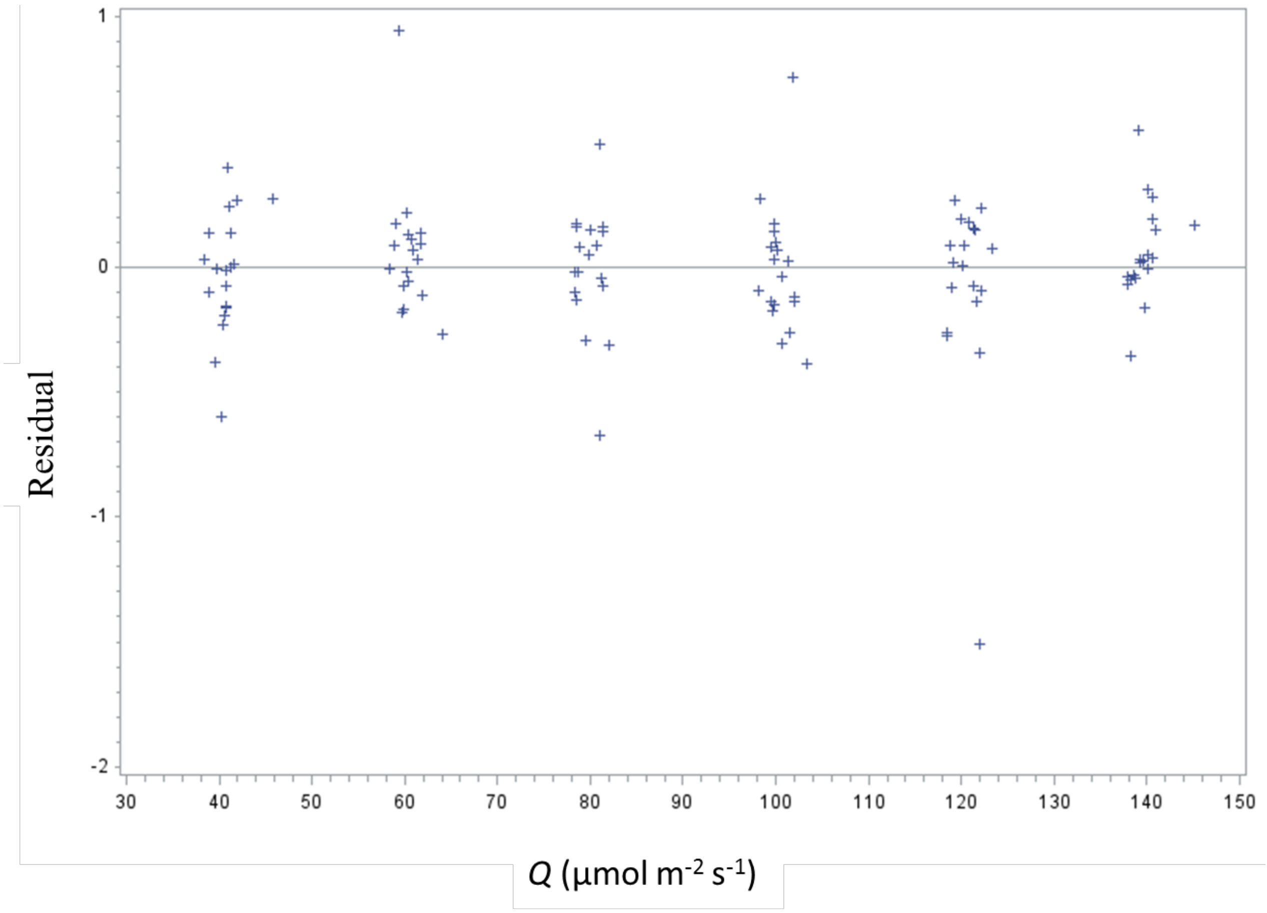

Fig. S2: Residuals of each linear regression of *A* vs. *Q* plotted against *Q* in lower canopy leaves of *Z. mays*

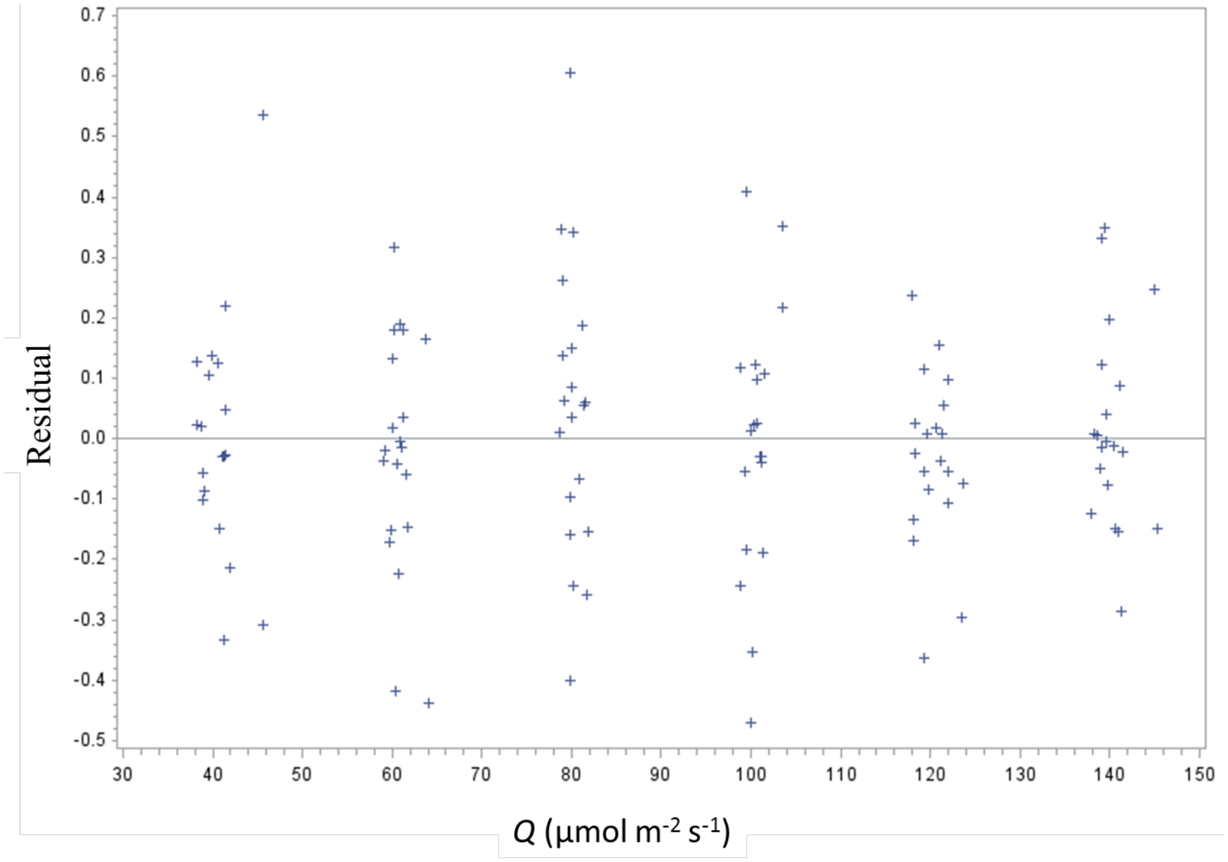

Fig. S3: Residuals of each linear regression of *A* vs. *Q* plotted against *Q* in upper canopy leaves of *Z. mays*

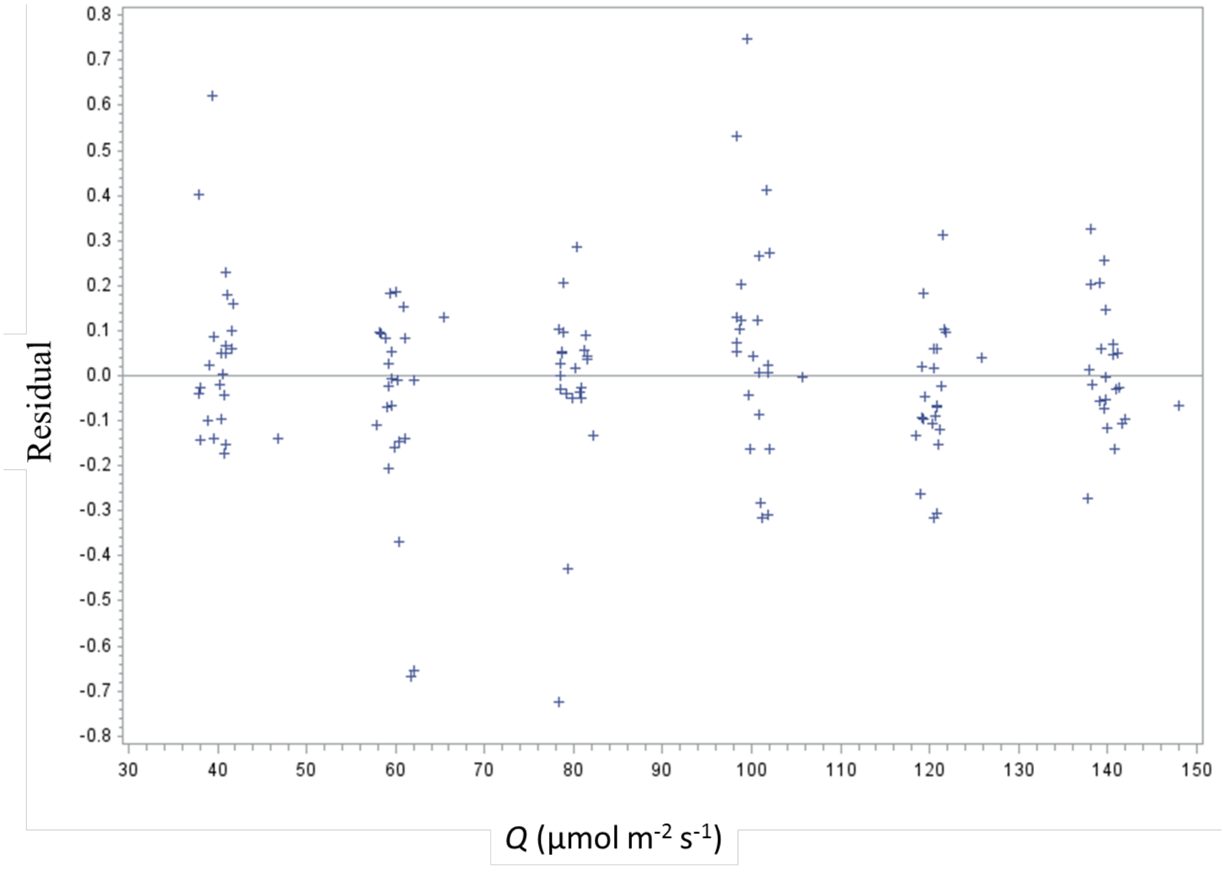

Fig. S4: Residuals of each linear regression of *A* vs. *Q* plotted against *Q* in lower canopy leaves of *M. x giganteus*

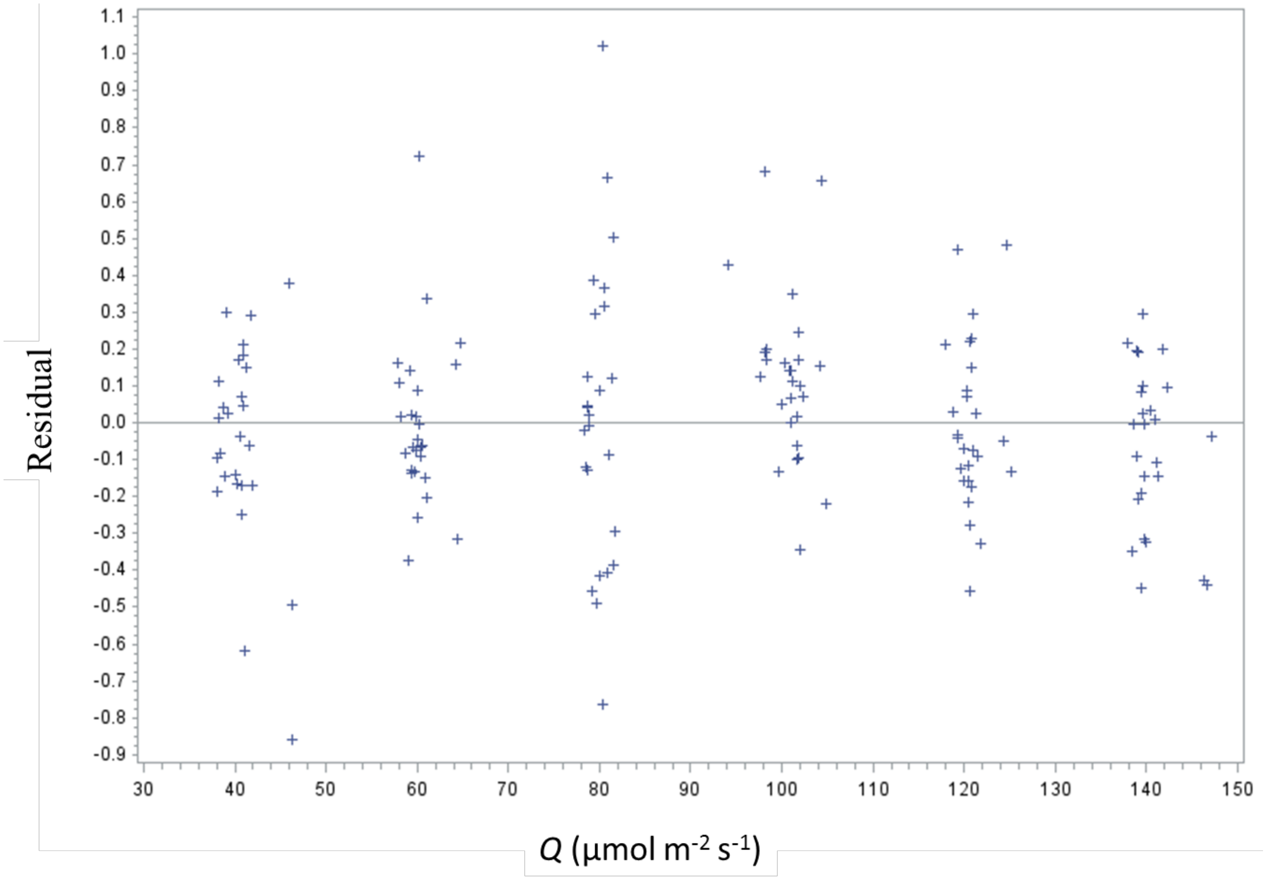

Fig. S5: Residuals of each linear regression of *A* vs. *Q* plotted against *Q* in upper canopy leaves of *M. x giganteus*
